# North Iowa Medical Society

**Published:** 1871-08

**Authors:** J. S. Green

**Affiliations:** Secretary


### Proceedings of Societies.

### NORTH IOWA MEDICAL SOCIETY.

This Society held its Eleventh Annual Meeting at the Office of Dr. Andros, in McGregor, on Wednesday, June 7th. Dr. F. Andros, President, in the chair.

Roll called, and found present, Drs. F. Andros, H. H. Clark, W. C. Lewis, J. S. Green, J. T. H. Scott, D. W. Chase, W. H. Earl, and L. Brown, Jun.

Minutes of last meeting read and approved.

Drs. J. W. Curtis, of Decorah; A. B. Hanna, of Elkader; E. R. Zeigler and S. E. Robinson, of West Union; were duly elected members of the Society.

Communications were received from Drs. N. H. Knowles, of Hesper, and Lewis L. Hayeltine, of New York City.

The following resolutions were adopted:

*Whereas,* in the conscientious discharge of duty, Dr. Lewis L. Hayeltine has removed from a sphere of usefulness which he has sucessfully exercised for the benefit of the public and to the honor of the profession; and, *Whereas,* from his first connection with this Society to its close he was a faithful and efficient member; therefore,

*Resolved,* That we, the Members of the North Iowa Medical Society, hereby express regret at his loss, and unabated interest in his future welfare.

*Resolved,* That the Secretary be authorized to place his name upon the list of Honorary Members, and that a copy of our proceedings be at all times sent him.

The President, Dr. Andros, read his annual address—a well-written essay upon anæsthetics—which brought forth much discussion from several members. The discussion to be continued at the next meeting.

Dr. Curtis made a very lengthy verbal report upon the value of the thermometer in the diagnosis and prognosis of disease.

Dr. H. H. Clark, of McGregor, reported the case of a patient who had been subject to epileptic fits about five years, the attacks becoming gradually more frequent, until she had from three to six each week. Was called to see her about Aug. 24th, 1870. Prescribed potassa bromidi, grs. 30, four times a day, after which she had no more fits, but gradually passed into a comatose condition, death relieving her suffering, Sept. 27th. The result of *post mortem* was presented to the Society, in the shape of a fibro-cartilaginous tumor, weighing 3 oz., with a small point about ¼in. in diameter, indicating the point of attachment, which had been attached to the squamous portion of the right temporal bone.

There was a lengthy report upon the effects and use of the hydrate of chloral.

Dr. Brown had used it in two cases of puerperal convulsions, and in one case of partial mania, caused by the poison of tobacco, applied in the form of a poultice, to a carbuncle. All three cases yielded promptly to it.

Dr. Scott had used it in one case, and Dr. Green in many cases of protracted and difficult labor, with the best success—causing relaxation, and sleep between pains, more than any other drug in use.

Dr. Chase reported a case, a patient of his—practising physician—who, upon his own responsibility, took over an ounce at a time, without producing any deleterious effects, only, thirty hours profound sleep.

Dr. Scott reported a very interesting, and in some respects unusual, case of strangulated scrotal hernia, in a young man whom he and Dr. Andros operated on, and found a large part of the omentum mortified, which they amputated, and ligated the stump, leaving one end of the ligature fastened external to the wound; but from some cause it got loose, and was drawn up into the abdomen. Seven weeks after an abscess appeared in the umbilical region, which discharged a few ounces of pus and the ligature. The patient made a rapid recovery.

Many interesting cases were verbally reported, and opinions given by different members.

The Constitution was changed, so as to admit Howard County in the territory of the Association.

On motion of Dr. Robertson, the President was directed to appoint a Committee of one to report upon Surgery, Obstetrical Practice, and New Remedies, to report at the next Annual Meeting.

The following officers were elected for the ensuing year:—

*President*—W. C. Lewis, of Clermont.

*Vice-President*—Luther Brown, of Postville.

*Secretary*—J. S. Green, of Postville.

*Treasurer*—H. H. Clark, of McGregor.

*Censors*—J. S. Green, J. T. H. Scott, and H. H. Clark.

*Delegates to the American Medical Association*—F. Andros and J. W. Curtis.

*Essayists*—Drs. F. Andros, J. W. Curtis, and H. B. Curtis.

The Secretary was authorized to make credentials to any member who would attend the State Medical Society.

Adjourned, to have the next Annual Meeting at Postville, on the first Wednesday of June, 1872; also, a Semi-Annual Meeting at Decorah, on Wednesday, October 25th, 1871.

All regular physicians in good standing are requested to attend.

J. S. Green, M.D., *Secretary.*

---

